# Keratoderma in Systemic Lupus Erythematosus: An Unusual Cutaneous Manifestation of Disease?

**DOI:** 10.7759/cureus.30736

**Published:** 2022-10-26

**Authors:** Jayashankar CA, Prakash Bhanu, Sravanthi S Dandu, Amey Joshi, Hiba Salam, Lenora S Barre, Sree N Nallamothu

**Affiliations:** 1 Internal Medicine, Vydehi Institute of Medical Sciences and Research Centre, Bengaluru, IND; 2 Dermatology, Vydehi Institute of Medical Sciences and Research Centre, Bengaluru, IND; 3 General Medicine, Vydehi Institute of Medical Sciences and Research Centre, Bengaluru, IND; 4 General Medicine, Manipal Hospitals, Bengaluru, IND

**Keywords:** lupus, sle flare, autoimmune, keratoderma, systemic lupus erythematosus

## Abstract

Systemic lupus erythematosus (SLE) is a multi-organ autoimmune disease, with the skin being the second most affected organ after the joints. We present a unique case of a 44-year-old female who presented with an acute flare of SLE and the concurrent onset of keratoderma on both lower limbs. She presented with high-grade fever, arthralgia, and generalized edema of four months duration. A general physical examination revealed pallor and scaly hyperpigmented plaques on both lower limbs, which was confirmed to be keratoderma on histopathological examination. Blood investigations revealed pancytopenia, elevated erythrocyte sedimentation rate (ESR) and C- reactive protein (CRP), and positive titers for anti-nuclear antibody (ANA) and anti-Po ribosomal P proteins (RPP) antibodies. Immunosuppressive medications and topical keratolytics were used to treat her successfully. Post medical management, she showed significant improvement in her symptoms. On follow-up, the patient had a complete resolution of the symptoms and remained well. This case demonstrates keratoderma as a rare incidental finding in a patient with SLE flare. Understanding SLE's various cutaneous manifestations are critical for holistically diagnosing and treating the disease.

## Introduction

Systemic lupus erythematosus (SLE) is a complex autoimmune disease that affects multiple organs [1-2]. The disease primarily affects the joints and skin, with nephropathy, malar rash, photosensitivity, and serositis being the most common manifestations [1,3]. Skin lesions in SLE are classified as lupus-specific skin lesions, including malar rash, photosensitive dermatitis, discoid rash, subacute cutaneous lupus erythematosus, and lupus non-specific conditions, which include bullous lesions, pyoderma gangrenosum, and erythema multiforme [1,4]. SLE skin lesions can cause invocational handicaps and significant morbidity in patients by causing scarring, disfigurement, and alopecia [1,5]. Understanding SLE's various cutaneous manifestations is critical for holistically diagnosing and treating the disease.

Keratoderma is a keratinizing disorder that is characterized by hyperkeratotic thickening of the palms and soles [6-9]. Mutations in the gene-encoding proteins involved in the keratinization process, such as keratins, desmosomes, loricrin, cathepsin C, and gap junction proteins, are responsible for this pathology [6-9]. This condition necessitates aggressive treatment and may necessitate advanced wound care management [8]. Although keratoderma has been identified to arise from acquired causes and in genetically predisposed individuals, there is limited literature on keratoderma in SLE patients [8,10,11]. We present a case of an SLE flare with an incidental finding of recent onset keratoderma on both legs.

## Case presentation

**Figure 1:**
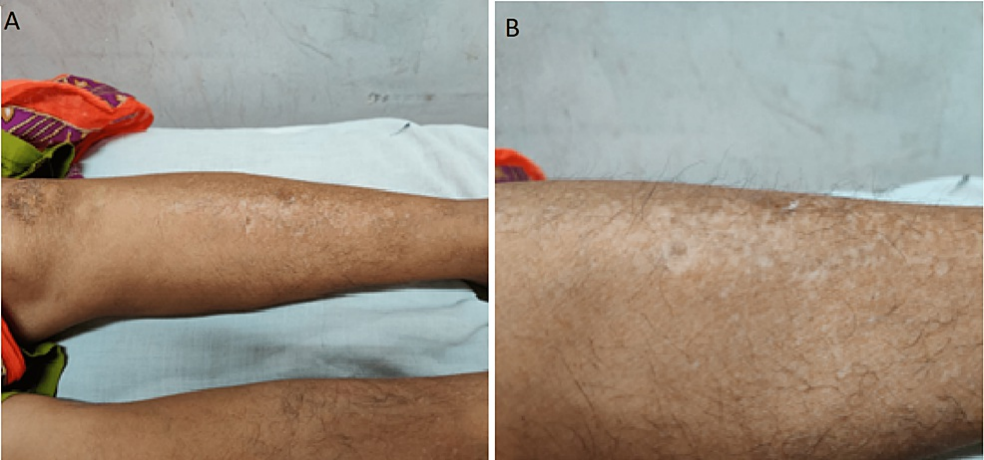
Gross image of skin lesion on legs (A), (B): Scaly hyperpigmented plaques on both lower limbs, suggestive of keratoderma

A 44-year-old female presented with recurrent episodes of high-grade fever for four months. History was significant for four months of arthralgia in the knee, shoulder, elbow, and wrist joints and generalized edema. The patient did not report loss of appetite, haematuria, breathlessness, chest pain, and abdominal pain. The patient was diagnosed with SLE four months before the current presentation by a rheumatologist and was prescribed oral methotrexate, prednisolone, and etoricoxib. However, the patient was non-adherent to these medications. The general physical examination revealed conjunctival pallor and scaly hyperpigmented plaques on both lower limbs (Figure 1).

The patient was febrile (104.2 F) and had tachycardia. Respiratory, cardiovascular, per-abdominal, locomotor, and neurological examinations were unremarkable. Laboratory investigations revealed pancytopenia, elevated ESR, and C- reactive protein (CRP). Renal and liver function tests, blood glucose, coagulation profile, thyroid profile, serum electrolytes, urine routine chemistry, and microscopy were within the normal range. Table 1 gives the details of the relevant paraclinical studies.

**Table 1:** Most representative paraclinical studies at admission IFA: Indirect Immunofluorescence Assay

Paraclinical studies	Patient values	Normal values
White Blood Cell Count (10^3^uL)	3.9	4.0-11.0
Hemoglobin (g/dl)	6.8	11.5-15.0
Platelet Count (10^3^uL)	140	150-410
Absolute Neutrophil Count (10^3^uL)	2.4	2-7
Absolute Lymphocyte Count (10^3^uL)	1.2	1-3
Absolute Monocyte Count (10^3^uL)	0.2	0.2-1
Absolute Eosinophil Count (10^3^uL)	0	0.02-0.50
Absolute Basophil Count (10^3^uL)	0	0.02-0.10
Serum complement C3 (mg/dl)	5.9	79-152
Serum complement C4 (mg/dl)	17.1	16-38
Aspartate Transaminase (IU/L)	159	13-35
Alanine Transaminase (IU/L)	57	7-35
Serum Iron (mcg/dL)	22	50-170
Serum C-Reactive Protein (mg/L)	4.4	0-<0.8
Erythrocyte Sedimentation Rate (ESR) (mm/hr)	61	0-15
Anti-Nuclear Antibody (IFA)	+++	Negative
Anti-dsDNA Antibody	Negative	Negative
Anti-Ribosomal P Protein Antibody	Positive	Negative
Serum Ferritin (ng/mL)	>1500	10-120
Serum Transferrin (mg/dL)	300	200-400
Mean Corpuscular Volume (fL)	82.2	83.0-101.0
Mean Corpuscular Hemoglobin Concentration (g/dL)	31.6	31.5-34.5
Peripheral Blood Smear	Microcytic hypochromic anaemia of severe degree with pancytopenia	
Blood Culture	No growth after five days of culture	
Urine Analysis	Normal	
Urine Culture	No growth	

**Figure 2:**
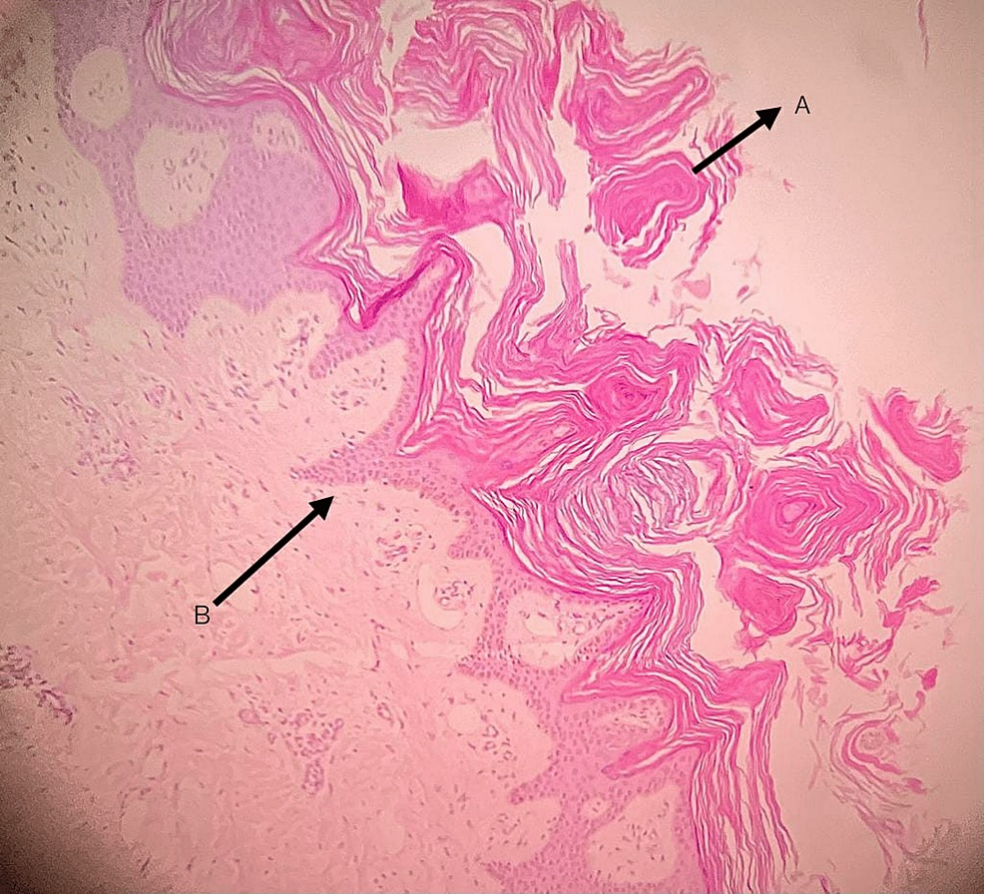
Histopathology of skin lesion on leg Histopathology image of skin biopsy from the scaly hyper-pigmented plaques showing irregularly thickened epidermis with florid acanthosis, (B) lamellar hyperkeratosis, and (A) focal follicular plugging suggestive of keratoderma.

Serum C3 complement levels were low (51.9 mg/dl), and ANA was positive. ANA-15 screen was also positive for anti-Po RPP antibodies. The direct Coombs test was negative. ECG, echocardiography, chest x-ray, and ultrasonography of the abdomen and pelvis revealed no significant abnormality. Skin biopsy of the scaly hyperpigmented plaques on lower limbs revealed an irregularly thickened epidermis with florid acanthosis, lamellar hyperkeratosis, and focal follicular plugging suggestive of keratoderma (Figure 2).

The patient was transfused with one unit of packed red blood cells for her anemia of chronic disease. She was administered oral prednisolone and etoricoxib for her joint pain. Her temperature spikes were controlled with acetaminophen infusions. Topical keratolytic agents (10% urea with salicylic acid and lactic acid cream) were used to manage the skin lesions. Post-medical management, she showed significant improvement in her symptoms. On discharge, the patient was advised oral prednisolone and topical keratolytic (10% urea with salicylic acid and lactic acid cream) and remained adherent to the medical management. On follow-up, the patient had a complete resolution of the symptoms and remained well. 

## Discussion

We present an unusual incidental progressive type of keratoderma in an SLE patient. Malar rash, discoid lupus erythematosus, photosensitivity, mucosal discoid lupus erythematosus, subacute cutaneous lupus erythematosus, alopecia, and lupus panniculitis are all cutaneous manifestations of SLE [1,4,12]. Although rare, keratoderma has been reported as a co-existent or incidental finding in patients with SLE in a few studies [1,4,8,10-12]. In a Chittagong cross-sectional survey, palmoplantar keratoderma was found in 2.5% of SLE patients [10]. Keratoderma is a keratinizing disorder caused by genetic or acquired causes such as systemic diseases, infections, and cancer [6-9,13]. Keratoderma can be focal, striate, punctate, or diffuse [6]. Hereditary keratoderma is inherited in an autosomal dominant or recessive manner and is caused by mutations in the gene encoding proteins involved in the keratinization process [6-9]. Other causes of keratoderma include systemic disease, malnutrition, drug-related infections, malignancy, and dermatoses [13]. Hypothyroidism, chronic lymphedema, myxoedema, and various circulatory disorders such as acrocyanosis and livedo reticularis are systemic diseases associated with keratoderma [13]. A study from Michigan, United States, reported two SLE patients with ulcerative plantar keratoderma, one with diffuse desquamation over the entire plantar surface and the other with focal lesions over the weight-bearing surfaces [8]. The management of keratoderma involves the investigation of the underlying etiology and its treatment [13]. In the absence of an underlying cause, the current treatment options include topical keratolytics such as urea, salicylic acid, and lactic acid, topical retinoids and corticosteroids, physical debridement, and topical psoralen with ultraviolet A (UVA) [13]. In the present case, our patient presented with scaly hyperpigmented plaques with histopathological features consistent with keratoderma in the lower limbs and was successfully treated with topical keratolytic (10% urea with salicylic acid and lactic acid).

The relationship between keratoderma and SLE has not been established due to the very few case reports published [8,10,11]. This case of keratoderma in SLE necessitates further research to determine the association between the two pathologies if any. 

## Conclusions

This case illustrates the unusual occurrence of keratoderma of the legs in an SLE patient. The management of keratoderma requires a multifaceted approach with treatment focused on possible underlying etiologies and topical therapies. Treatment of the SLE flare in the present case with immunosuppressants and the topical use of urea with salicylic acid resulted in the gradual improvement of the patients’ keratoderma. Other topical agents like lactic acid, topical retinoids and corticosteroids, physical debridement, and topical psoralen with UVA can be considered in treating keratoderma. However, the possible association of keratoderma in SLE remains to be established.
